# Long-Term Follow-Up of Catheter Ablation for Premature Ventricular Complexes in the Modern Era: The Importance of Localization and Substrate

**DOI:** 10.3390/jcm11216583

**Published:** 2022-11-06

**Authors:** Simone Gulletta, Alessio Gasperetti, Marco Schiavone, Gabriele Paglino, Pasquale Vergara, Paolo Compagnucci, Caterina Bisceglia, Manuela Cireddu, Nicolai Fierro, Giuseppe D’Angelo, Simone Sala, Lorenzo Rampa, Michela Casella, Patrizio Mazzone, Antonio Dello Russo, Giovanni Battista Forleo, Paolo Della Bella

**Affiliations:** 1 Department of Arrhythmology, San Raffaele Hospital, 20132 Milan, MI, Italy; 2 Cardiology Unit, Luigi Sacco University Hospital, 20157 Milan, MI, Italy; 3 Division of Cardiology, Department of Medicine, Johns Hopkins University, Baltimore, MD 21218, USA; 4 Department of Systems Medicine, University of Rome Tor Vergata, 00133 Rome, RM, Italy; 5 Cardiology and Arrhythmology Clinic, University Hospital Umberto-I-Salesi-Lancisi, 60123 Ancona, AN, Italy

**Keywords:** catheter ablation, premature ventricular complexes, arrhythmic recurrences, clinical outcomes, complications

## Abstract

**Background:** Large-scale studies evaluating long-term recurrence rates in both idiopathic and non-idiopathic PVC catheter ablation (CA) patients have not been reported. **Objective:** To evaluate the efficacy and safety of idiopathic and non-idiopathic PVC CA, investigating the predictors of acute and long-term efficacy. **Methods:** This retrospective multicentric study included 439 patients who underwent PVC CA at three institutions from April-2015 to December-2021. Clinical success at 6 months’ follow-up, defined as a reduction of at least 80% of the pre-procedural PVC burden, was deemed the primary outcome. The secondary aims of the study were: clinical success at the last available follow-up, predictors of arrhythmic recurrences at long-term follow-up, and safety outcomes. **Results:** The median age was 51 years, with 24.9% patients being affected suffering from structural heart disease. The median pre-procedural PVC burden was 20.1%. PVCs originating from the RVOT were the most common index PVC observed (29.1%), followed by coronary cusp (CC) and non-outflow tract (OT) LV PVCs (23.1% and 19.0%). The primary outcome at 6 months was reached in 85.1% cases, with a significant reduction in the 24 h% PVC burden (−91.4% [−83.4; −96.7], *p* < 0.001); long-term efficacy was observed in 82.1% of cases at almost 3-year follow-up. The presence of underlying structural heart disease and non-OT LV region origin (aHR 1.77 [1.07–2.93], *p* = 0.027 and aHR = 1.96 [1.22–3.14], *p* = 0.005) was independently associated with recurrences. **Conclusion:** CA of both idiopathic and non-idiopathic PVCs showed a very good acute and long-term procedural success rate, with an overall low complication. Predictors of arrhythmic recurrence at follow-up were underlying structural heart disease and non-OT LV origin.

## 1. Introduction

In common clinical practice, premature ventricular complexes (PVCs) are one of the most frequently encountered cardiac arrhythmia. Besides being related to potentially debilitating symptoms, PVCs are common in patients with LV dysfunction and are often organized as runs of non-sustained ventricular tachycardia (NSVT). PVCs may represent the consequence or the cause of LV dysfunction or dilation, with a burden of >24% that has been shown to be independently associated with PVC-induced cardiomyopathy [1]. Single-center studies have demonstrated that catheter ablation (CA) is superior to pharmacological therapy in decreasing PVC burden and improving cardiac function [2,3], but these findings are mostly based on small-sample-size studies, as summarized in a meta-analysis from Zang et al. [4] on this topic. This report has outlined how CA of frequent PVCs improves cardiac function, especially in patients with LV dysfunction. The safety and efficacy of CA has then been confirmed in one large-scale trial from Latchamsetty et al., retrospectively enrolling 1185 idiopathic PVC patients [5]. Nevertheless, Holter ECG monitoring at follow-up was not available in all patients in this study; thus, complete data to assess procedural efficacy were reported in 490 patients. It is known that the highest success rate, along with the lowest peri-procedural complication rate, is observed in patients with PVCs originating from the RVOT, followed by PVC arising from the LVOT. However, few data on the relationship between specific PVC sites of origin and CA clinical outcomes are currently available, with no specific report addressing CA of PVCs arising from the coronary cusps (CC). Besides CC, data on the clinical outcomes of PVC CA arising from other non-OT locations are scarce as well, the main reason being that those are typically challenging locations, such as the LV summit and papillary muscles, due to their anatomy. The LV summit is the highest portion of the LV epicardium, being located near the left main coronary artery, and may account for up to 14.5% of LV ventricular arrhythmias (VAs), according to some reports [6]. Moreover, the papillary muscles are also a well-known source of VAs in both structurally normal and abnormal hearts, with PVCs arising from this location potentially playing a role in triggering even ventricular fibrillation [7]. Therefore, the purpose of this real-world study was to evaluate efficacy, clinical outcomes, and procedural complications of both idiopathic and non-idiopathic PVC CA, stratified for sites of origin, and to determine the predictors of acute and long-term efficacy.

## 2. Materials and Methods

### 2.1. Patient Population

All consecutive patients undergoing catheter-based PVC ablation at three tertiary centers for cardiac electrophysiology (EP) (IRCCS Ospedale San Raffaele, Milan, Italy; Luigi Sacco University Hospital, Milan, Italy; University Hospital “Lancisi-Salesi”, Ancona, Italy) from April 2015 to December 2021 were retrospectively enrolled in this observational multicenter study. The study was approved by the Local Institutional Review Board and complies with the Declaration of Helsinki.

### 2.2. Procedural Details

All antiarrhythmic drugs were withdrawn at least 3 days prior to the procedure. Three-dimensional electroanatomical mapping and pace mapping with Carto System (Biosense Webster Inc., Irvine, CA, USA) or EnSite NavX Endocardial Solutions System (St. Jude Medical, Inc., St. Paul, MN, USA) were used to detect the earliest ectopic ventricular activation. Activation mapping was used if PVCs occurred frequently enough, otherwise the CA procedure relied mostly on pace mapping. If few PVCs were observed at baseline, and there was a consistent documentation of significant pre-procedural PVC burden, intravenous administration of isoproterenol and/or programmed ventricular stimulation was performed to try to induce ventricular arrhythmias and then compared to the baseline PVC. When endocardial mapping was not satisfactory, a mapping catheter was advanced via the CS to obtain an epicardial map from the great cardiac vein (GCV) or anterior interventricular vein (AIV). An irrigated-tip ablation catheter was used to perform radiofrequency (RF) lesions. Catheter choice and the use of robotic magnetic navigation system was left to the operator’s discretion, as well as the use of ablation index to guide CA. The need to perform coronary angiography during CA for PVC arising from the LV summit was also left to the electrophysiologist’s discretion. Whenever ablation from the GCV was needed, RF was delivered for a maximum of 20 W per 30 s, and RF was stopped earlier in case of sudden drops of impedance and/or PVC sudden interruption. For left-sided procedures, either via a transeptal access or via a retroaortic approach, heparin boluses were administered to maintain an activated clotting time ≥300 s during the whole procedure. After ablation, the “watching time” was set at 30 min to ensure procedural success, which was defined as the disappearance or the non-inducibility of the targeted PVC.

### 2.3. Data Collection and Follow-Up Strategies

All data were collected into a centralized, anonymized spreadsheet. Demographics and cardiovascular comorbidities were assessed for all patients enrolled in the study. Additionally, a 12-lead ECG capturing the index PVC both in precordial and peripheral leads and a 24-ECG Holter retrieved in all patients prior to the index ablation procedure were analyzed. For PVC characterization, the following data were collected: 24 h% burden; number of couplets/24 h; number of triplets; PVC QRS duration (in ms); and PVC coupling cycle (in ms). In accordance with PVC morphology, axis, and final ablation site, PVC origin was determined. PVCs were then grouped into 5 classifier groups, as follows: (a) CC PVC; (b) left ventricular outflow tract (LVOT) PVC; (c) non-outflow tract LV (non-OT LV) PVC; (d) right ventricular outflow tract (RVOT) PVC; (e) non-outflow tract RV (non-OT RV) PVC. Those 5 classifiers were used to group study patients in the study sub-cohorts. After discharge from the index procedure, follow-up strategies were at the discretion of a single physician, with most patients being first evaluated at 6 months’ follow-up (first follow-up visit). The first evaluation always included a full cardiovascular examination, a 12-lead ECG and a 24 h Holter ECG; otherwise, patients were excluded from the current analysis. Subsequently, all patients were usually evaluated every 12 months thereafter, including a 24 h Holter ECG. Patients who were followed-up for less than 6 months were excluded from this study as well. 

### 2.4. Variable Definitions and Study Outcomes

A patient was deemed to have a structural heart disease in the presence of a diagnosed structural cardiomyopathy (such as ischemic heart disease, myocarditis, non-ischemic cardiomyopathy, and moderate to severe valvular heart disease). Chronic kidney disease (CKD) was defined as a glomerular filtration rate (GFR) < 60 mL/min. The primary outcome of the study was the clinical success of CA at 6 months of follow-up from the index procedure. Consistently with previous studies on this topic [5], the clinical success of CA was defined as a reduction of at least 80% in the pre-procedural 24 h PVC burden at follow-up Holter ECGs. The secondary aims of the study were: (a) to report the rate of clinical success of CA at the last available follow-up assessment; (b) to report the predictors of arrhythmic recurrences at long-term follow-up; (c) safety outcomes deemed as major post-procedural complications, including: vascular access issues (vascular hematoma, pseudoaneurysms, and atrioventricular fistula), pericardial effusion, pericardial tamponade requiring or not requiring pericardiocentesis and/or cardiac surgery, and transient or permanent conduction system damage (i.e., resulting in atrioventricular block).

### 2.5. Statistical Analysis

All analyses were performed using STATA v.13 (StataCorp LLC, 4905 Lakeway Drive, College Station, TX, USA). Normality of distribution of continuous variables was tested using a Shapiro–Wilk test. Normally and non-normally distributed variables were reported as mean standard deviation (s.d.) and as median (interquartile range (IQR)), as appropriate. Categorical variables were reported as count (percentage). Comparisons of continuous variables among different groups were performed through one-way analysis of variance (ANOVA) or through a Kruskal–Wallis test, according to distribution. Pairwise comparisons between non-normally distributed variables were performed using a pairwise Wilcoxon test. Comparisons of categorical variables were performed using a chi-squared or a Fisher exact test, as appropriate. Survival from arrhythmic recurrence was reported graphically using Kaplan–Meier (KM) curves. Differences between KM curves were assessed through a log-rank test. Pre-specified predictors of arrhythmic recurrence were tested using a univariate Cox regression model and their association to outcomes reported with hazard ratios (HRs). A multivariate Cox regression was then fitted, including all predictors which were significantly associated with the outcome at univariate analysis. Adjusted hazard ratios were reported (aHRs). A two-tailed **α** < 0.05 was considered statistically significant through the study.

## 3. Results

### 3.1. Patient Population

A total of 439 patients were enrolled in the study. The median age at index procedure was 51 [36–62] years and 65.5% of patients were male. The arrhythmic burden at the time of procedure was elevated, with the median% PVC burden being 20.1% [11.6–34.5] and patients presenting a median of 377 [128–668] PVC couplets and 57 PVC triplets at the pre-procedural 24 h Holter ECG assessment. The origin of index PVC was distributed as follows: CC *n* = 101 (23.1); LVOT *n* = 70 (16.0); non-OT LV *n* = 83 (19.0); RVOT *n* = 127 (29.1); and non-OT RV *n* = 56 (12.8). The median PVC QRS duration was 148 [136–157] ms and the PVC coupling cycle was 478 [427–538] ms. A structural heart disease was present in a quarter of the study cohort (*n* = 109, 24.9%), with ischemic cardiomyopathy being the most common etiology (*n* = 44, 10.0%). The complete characteristics of the study population are reported in Table 1.

### 3.2. Procedural Data

Among the 439 patients enrolled, 354 patients underwent PVC ablation using contact-force Biosense Webster Inc. (Irvine, CA, USA) catheters (mostly Thermocool SmartTouch^®^ catheters), 74 using St. Jude Medical (St. Paul, MN, USA) catheters (mostly the FlexAbility ablation catheter), and 11 using the Stereotaxis Niobe^®^ Robotic Magnetic Navigation System (St. Louis, MO, USA) with catheters based on their contact force systems. Ablation-index-guided procedures were based on Thermocool SmartTouch^®^ catheters. The overall procedural time and fluoroscopy time were 118.9 ± 45.5 min and 30 [20–35] min, respectively, with an average of 317 [180–570] s of RF time. Peri-procedural success was high (97.5%) and complications uncommon (10/439, 2.2%; *n* = 1 pericardial tamponade; *n* = 4 pericardial effusion; and *n* = 5 vascular access complications). Table 2 reports the overall cohort and by their PVC localization peri-procedural characteristics.

### 3.3. Study Outcomes and Outcome Predictors

**Figure 1:**
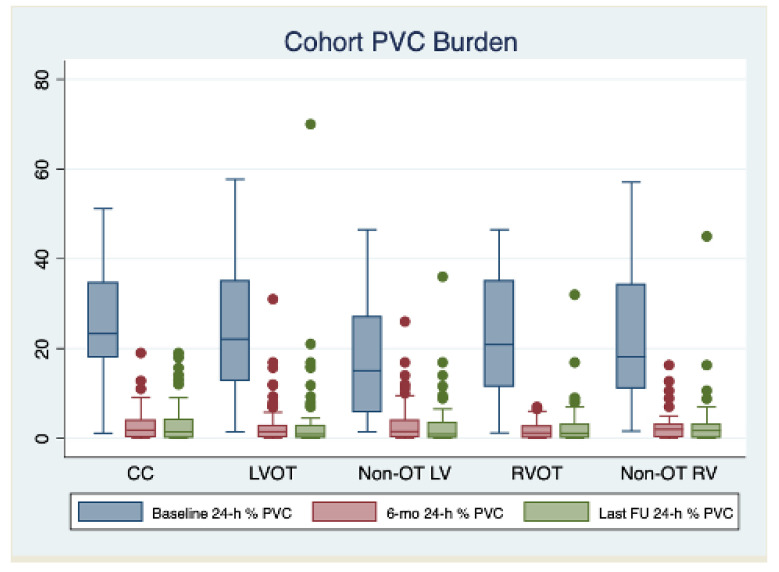
Changes in 24 h% burden over time stratified by PVC localization. Abbreviations: CC: coronary cusps, LV: left ventricle, LVOT: left ventricular outflow tract, OT: outflow tract, RV: right ventricle, RVOT: right ventricular outflow tract.

**Figure 2:**
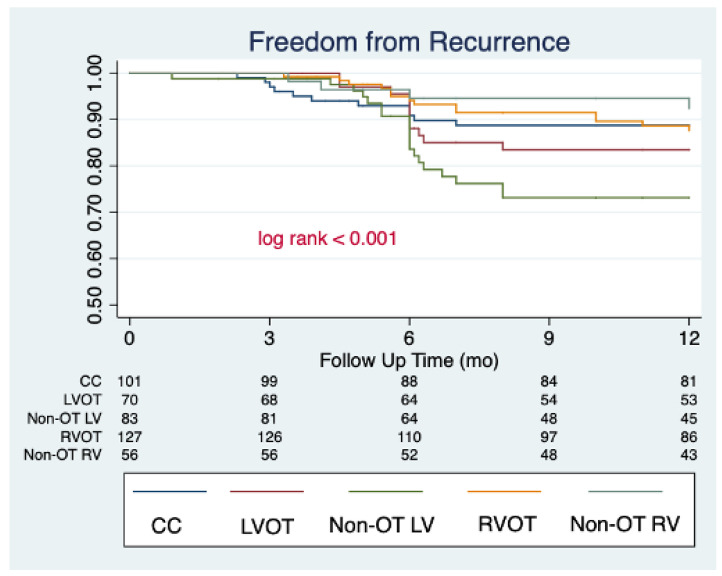
Clinical success of PVC catheter ablation stratified by arising zones, graphically displayed by Kaplan–Meier curves.

The median follow-up of the study was 34 [24–40] months. All patients completed their first follow-up visit. At first follow-up post index procedure, a significant reduction in the PVC burden was observed (median 24 h% burden: 1.5 [0.3–3.0]; median % reduction from baseline: −91.4% [−83.4; −96.7], *p* < 0.001), with 372 (85.1%) patients being free from arrhythmia recurrences. A significant decrease in 24 h% PVC burden was observed independently from PVC localization, but the rate of freedom from recurrences was significantly lower in patients with PVC originating from the non-OT LV area (70.0%), compared to the other localization (CC: 85.1%; LVOT: 88.1%; RVOT: 91.3%; non-OT RV: 92.9; *p* < 0.001). The 24 h% PVC burden and the rates of freedom from recurrences remained stable between the 6-month and last follow-up assessment in the entire cohort (85.1% vs. 82.1%; *p* = 0.230) and in the by-localization groups as well (CC: 88.1% vs. 83.2%, *p* = 0.321; LVOT: 81.4% vs. 80.0%, *p* = 0.834; 70.0% vs. 68.7%, *p* = 0.856; 92.9% vs. 88.2%, *p* = 0.415; 92.9% vs. 89.3%, *p* = 0.504). When evaluating anti-arrhythmic drug (AAD) therapy, the rate of patients on anti-arrhythmic drug (AAD) therapy was as follows: (1) 6-month follow-up: class IC AAD: 13.5%, sotalol 6.6%, amiodarone 5%, and without AAD 74.9%; (2) Last follow-up: class IC AAD 5.7%, sotalol 2.5%, amiodarone 1.8%, and without AAD 90%. The complete outcome data are reported in Table 3. The changes in 24 h% burden over time stratified by PVC localization are reported in Figure 1. By arising zones, freedom from arrhythmias is graphically displayed through the KM curves reported in Figure 2. Table 4 reports the clinical predictors of arrhythmic recurrences at long-term follow-up: the presence of structural heart disease, as well as the area of origin of PVC being the non-OT LV area, were associated with a lower freedom from arrhythmia at the last available follow-up. A higher PVC burden at the time of diagnosis was instead associated with a higher freedom from recurrences at follow-up. These three predictors remained significantly associated with arrhythmic recurrences even at multivariate analysis (aHR 1.96 [1.22–3.14], *p* = 0.005, aHR 1.77 [1.07–2.93], *p* = 0.027, and 0.96 [0.95–0.98], *p* < 0.001, respectively). On the other hand, the use of AAD during follow-up was not associated with the primary outcome at univariate analysis, as follows: HR 1.12 [0.67–1.87], *p* = 0.656.

## 4. Discussion

This study represents one of the largest experiences addressing the long-term outcomes of idiopathic and non-idiopathic PVC patients undergoing a catheter ablation procedure with modern technologies. The main results of the study may be summarized as follows:

PVC ablation is a common procedure, routinely performed in third-level electrophysiology centers. PVCs originating from the RVOT were the most common index PVC observed (29.1%), closely followed by CC and non-OT LV PVCs (23.1% and 19.0%).

The PVC ablation procedure had a very good acute procedural success rate (97.4%). A significant reduction in the 24 h% PVC burden was observed after PVC ablation (−91.4% [−83.4; −96.7], *p* < 0.001), with high rates of clinical success observed at 6 months and after an average of almost 3 years of follow-up (85.1% vs. 82.1%; respectively).

While a significant reduction in PVC burden was observed consistently across patients with PVC with different localizations, clinical success was significantly lower in patients with PVC originating from the non-OT LV regions (70.0% at 6 mo; 68.7% at last follow-up).

PVC origin from the non-OTF LV region (aHR 1.96 [1.22–3.14], *p* = 0.005) and the presence of an underlying structural heart disease (aHR 1.77 [1.07–2.93], *p* = 0.027) were independently associated with higher rates of arrhythmic recurrences during follow-up. 

CA of CC PVCs showed favorable outcomes at long-term follow-up (83.2%), ranging between RVOT and LVOT success rate (88.2% and 80.0%, respectively).

Complications were quite uncommon, being present in 2.2% of the entire cohort; these were mostly related to vascular access complications, with only one pericardial tamponade.

### 4.1. PVC Ablation and Clinical Outcomes

The long-term success rate of PVC ablation significantly varies, depending on the characteristics of the examined cohort. Indeed, in a meta-analysis from Zang et al. [4] the overall success rate ranges from 66% to 90%. The highest success rate was reached by Takemoto et al. [8], enrolling patients with frequent RVOT-PVC without evidence of structural heart disease, while Penela et al. [9] had the lowest procedural success, enrolling patients with LVEF < 50% of any etiology, with no particular exclusion criteria. These median success rates, along with differences mostly depending on the PVC arising zones and the underlying cardiac diseases, were subsequently confirmed in more recent trials, with Latchamsetty et al. [5] showing a 71–85% of long-term procedural success for idiopathic PVCs and Han et al. [10] having a 72.7% long-term success rate of PVC originating from periprosthetic aortic valve region ablations. Our findings are in line with these studies, with an overall success rate in our cohort (defined as a reduction of at least 80% of the pre-procedural 24 h PVC burden, according to these previous reports) of 85.1% at 6 months and of 82.1% at almost 3 years of follow-up, including patients with both idiopathic and non-idiopathic PVCs, our cohort being adequately heterogeneous with respect to other works. When analyzing the predictors of success of CA, the non-OT LV origin (LV summit, papillary muscles, and mitral valve left anterior/posterior fascicle) was the major determinant of PVC recurrence during follow-up (aHR 1.96 [1.22–3.14], *p* = 0.005), along with the presence of structural heart disease (aHR 1.77 [1.07–2.93], *p* = 0.027). On the other hand, the highest success rate was observed in patients with PVCs arising from the RV, either from the RVOT or from non-RVOT structures.

These results are surely not unexpected, but robust comparisons between groups are still lacking in the literature. Indeed, when analyzing predictors of adverse outcome in patients with frequent PVCs, in this large cohort study from Voskoboinik et al. [11], patients with structural heart disease were excluded from both validation cohorts. Moreover, Im et al. [12] showed that right ventricular PVC was among the unadjusted predictors of late PVC recurrence; however, the multivariate analysis failed to show that PVC site of origin was among the independent predictors for long-term success. Furthermore, patients with a history of structural heart disease were excluded. Several hypotheses may underpin our findings. First, papillary muscle PVCs are known to have a high recurrence rate, thus requiring longer RF delivery time and overall procedural times, although intracardiac echocardiography (ICE)-guided CA has been reported to be highly efficacious [13,14]. The non-systematic use of intracardiac echocardiography in all patients may partially explain the lower success rate of CA of non-OT LV PVCs in our cohort. Indeed, as described by Enriquez et al., ICE is crucial to guarantee adequate catheter–tissue contact and the correct orientation of the catheter tip during mapping and ablation. Moreover, ICE is essential to recognize increased echogenicity in the papillary muscles, identifying focal areas of scar that might highlight the site of arrhythmia origin, potentially corresponding to areas of low voltage and late potentials in sinus rhythm [15]. Second, LV summit and interventricular septum PVCs represent a major challenge as well, often requiring extensive mapping and subsequent ablation from the septal RVOT, coronary sinus, great cardiac vein, sub-aortic region, and sinuses of Valsalva, significantly prolonging the procedure and reducing the success rate even in the hands of experienced operators [16,17]. In our cohort, the need to perform coronary angiography during PVC ablation was left to the electrophysiologist’s discretion, and was mostly used during LV summit catheter ablations, before delivering RF from challenging locations, such as the GCV or whenever there was the strong suspicion of delivering RF near the coronary arteries, based on ICE imaging. Due the significant challenge that this location poses, if the earliest activation strategy failed in eliminating the target PVC, a “sandwich” ablation strategy, targeting the area from the opposite side, was attempted as a first-line choice. Whenever this protocol was also not effective in reaching the procedural success, and the managing clinician, in accordance with the patient, chose to perform a “redo” procedure, a subsequent alcohol ablation was attempted. Third, the “*liason*” between non-OT LV PVC and structural cardiac disease, which is, however, an independent predictor of PVC recurrence after CA, may also contribute to explaining our findings. Patients with underlying ischemic and non-ischemic cardiopathies are indeed known to be at the lowest rate of procedural success, as reported by Kazdri et al. [18] showing an acute procedural success of 60% in non-ischemic cardiomyopathies. 

Another important result of our study that should be mentioned in terms of procedural success is that patients with the highest pre-procedural burden are less keen to develop recurrences during follow-up. If this finding may initially appear counterintuitive, we believe that in these patients, mapping and precisely locating the target PVC is easier, therefore guaranteeing more efficient CA. Furthermore, it is evident that the rate of patients without AAD has significantly increased during follow-up, with most clinicians deciding to stop the AAD during follow-up. In our cohort, the use of AAD did not show an impact on the clinical success of PVC ablation, being not associated with the primary outcome of our study at univariate analysis.

Our results on CC PVC ablation should be briefly discussed as well. The overall procedural success rate of these PVC was found to be halfway between OT and non-OT RV and LV PVC, being achieved in 88.1% of patients at 6-month follow-up and 83.2% at long-term follow-up. Our success rate was overall slightly higher to that reported by other groups in the literature [5,10,19]. Indeed, differently from Latchamsetty et al. [5], showing that PVCs originating in the aortic cusps were at the highest risk of recurrence soon after papillary muscle PVCs, in our study, CC PVCs showed a higher success rate than LVOT PVC. Although lower power settings are often used in the aortic cusps to minimize the risk of complications and coronary artery injury [20], the consolidation of the RF lesion from both sides of the aortic cusps has allowed us to reach favorable clinical outcomes in those cases, without paying the price of a high number of complications. 

Our findings may have significant implications in clinical practice, especially when counseling patients about treatment options, always bearing in mind that the site of origin of PVC needs to be accurately evaluated with prediction algorithms [21], considering this significant influence on ablation outcomes. Moreover, an overall very favorable PVC burden reduction should encourage the managing clinician to offer CA as a robust treatment option in this scenario.

### 4.2. Ablation Index Use in PVC Ablation 

One of the most significant novelties of our study is represented by operators reporting the use of the ablation index (AI) module in 47.1% of the procedures. In recent days, the use of AI guidance for PVC ablation has been extensively investigated and a prospective study by Gasperetti et al. showed that AI guidance was superior to standard procedures in the management of idiopathic RVOT PVCs [22,23]. Validated procedural cut-offs, however, are currently limited to the RVOT area and no specific assessment in patients with an underlying structural heart disease has been performed. In this study, no specific cut-offs were given to operators, nor was a pre-determined AI protocol set in place. Both the use and the extent of reliance on AI was left completely to the choice of the individual operator. The use of an AI module in almost half of the procedures, however, shows how this multiparameter index has consistently taken part in the routine clinical practice of tertiary EP centers. Apart from a potential increase in procedural safety, one of the main advantages of AI guidance is the reduction in the inter- and intra-operator variability [24]. The standardization of cardiac EP procedures is appealing to the cardiac electrophysiology community as it helps increasing the reproducibility of results and the adherence to good clinical practices, and it reduces complications. AI is an already accepted tool, often used during PVC ablation. Its further investigation in the setting of specific structural heart substrates is, however, needed, as well as the definition of localization-specific AI thresholds.

### 4.3. Safety Endpoints

Major complications were quite uncommon in our cohort, with 2.2% suffering either from vascular access complications (50%) or from RF energy-related issues, with 1 (10%) pericardial tamponade, which was solved after pericardiocentesis. Despite our cohort being a priori at high risk of developing procedural complications, being—at least partially—represented by patients who also underwent non-OT LV ablation procedure, the overall number of life-threatening complications was overall low when compared to other clinical experiences. Indeed, it is generally believed that RF delivery near or on valvular structures in the heart can be performed safely [19], even though some cases have shown that ablation on or near the aortic valve can lead to valve dysfunction or coronary artery injury [25,26]. We believe that the high number of EP procedures performed in all the institutions involved in this study may be the main explanation of this finding.

### 4.4. Study Limitations

First, all the institutions participating in this study are tertiary referral EP centers, with all procedures being performed by experienced electrophysiologists; therefore, our clinical outcomes may not reflect outcomes achieved by less experiences by less-experienced operators. Second, despite reporting baseline ECG data and long-term Holter ECG, echocardiographic data were not available at follow-up, so eventual resolution of PVC-induced cardiomyopathy after CA could be not systematically assessed. Third, the decision on antiarrhythmic drug therapy interruption/continuation was left to every managing clinician during follow-up, without a pre-specified per-protocol recommendation. Fourth, the periodic patients’ evaluation with 24 h Holter ECG, that was performed yearly after the first follow-up visit (unless otherwise indicated), may have led to a partial underestimation of PVC recurrences, especially in asymptomatic patients. Last, the quantitative evaluation of symptom improvement after CA was not systematically performed according to a pre-specified clinical scale.

## 5. Conclusions

In our cohort from third-level EP centers, CA of both idiopathic and non-idiopathic PVCs showed a very good acute and long-term procedural success rate, with an overall low complication. Predictors of arrhythmic recurrence at follow-up were underlying structural heart disease and non-OT LV origin. CA of coronary cusp PVCs showed favorable long-term follow-up outcomes, ranging between RVOT and LVOT success rate.

## Figures and Tables

**Table 1:** Baseline Characteristics of the Study Cohort.

Study Population (*n* = 437)	
Age (years), median [IQR]	51 [36–62]
Male, *n* (%)	286 (65.5)
Diabetes, *n* (%)	42 (26.9)
HF, *n* (%)	26 (6.0)
Hypertension, *n* (%)	87 (19.9)
CKD, *n* (%)	10 (2.9)
AF, *n* (%)	23 (5.3)
Sport practice, *n* (%)	57 (13.0)
LVEF (%), mean ± s.d.	54.1 ± 9.9
Structural heart disease, *n* (%)	109 (24.9)
PVC localization	
CC, *n* (%)	101 (23.1)
LVOT, *n* (%)	70 (16.0)
Non-OT LV, *n* (%)	83 (19.0)
RVOT, *n* (%)	127 (29.1)
Non-OT RV, *n* (%)	56 (12.8)
PVC QRS length, median [IQR]	148 [136–157]
PVC coupling, median [IQR]	478 [427–538]
Arrhythmic burden	
24 h PVC burden %, median [IQR]	20.1 [11.6–34.5]
N of Couplets, median [IQR]	377 [128–668]
N of Triplets, median [IQR]	57 [24–92]
Palpitations, *n* (%)	274 (62.7)
Pharmacological therapy	
Beta-blockers, *n* (%)	216 (49.4)
Class Ic, *n* (%)	140 (32.0)
Class III, *n* (%)	110 (25.2)

Abbreviations: AF: atrial fibrillation, CC: coronary cusp, CKD: chronic kidney disease, HF: heart failure, LVEF: left ventricular ejection fraction, LVOT: left ventricular outflow tract, PVC: premature ventricular complex, RVOT: right ventricular outflow tract.

**Table 2:** Procedural Characteristics.

	Overall (*n* = 437)	CC (*n* = 101)	LVOT (*n* = 70)	Non-OT LV (*n* = 83)	RVOT (*n* = 127)	Non-OT RV (*n* = 56)	*p* Value
Procedure time (min), mean ± s.d.	118.9 ± 45.5	107.5 ± 42.0	124.7 ± 55.0	129.1 ± 44.3	117.6 ± 41.9	120.0 ± 48.1	**0.043**
Fluoroscopic time (min), median [IQR]	30 [20–35]	25 [20–30]	28 [15–40]	30 [25–45]	30 [20–35]	20 [15–35]	**0.002**
Radiofrequency time (s), median [IQR]	317 [180–570]	279 [180–600]	345 [179–575]	378 [200–721]	300 [188–515]	280 [176–580]	0.573
Power (W), mean ± s.d.	42.1 ± 10.6	46.9 ± 9.9	49.2 ± 8.0	43.8 ± 9.9	35.2 ± 8.4	37.3 ± 9.4	**<0.001**
Ablation-index-guided, *n* (%)	206 (47.1)	51 (50.5)	21 (30.0)	37 (44.6)	77 (60.6)	20 (35.7)	**<0.001**

Abbreviations: CC: coronary cusps; F.U.: follow-up, LVOT: left ventricular outflow tract, OT: outflow tract, RVOT: right ventricular outflow tract. Statistically significant values have been indicated in bold.

**Table 3:** Study Outcome.

	Baseline	6-mo F.U.			Last F.U.		
	Burden % Median [IQR]	Burden % Median [IQR]	*p*	Procedure Success (%)	Burden % Median [IQR]	*p*	Procedure Success (%)
Overall Cohort (*n* = 437)	20.1 [11.6–34.5]	1.5 [0.3–3.0]	**<0.001**	372 (85.1)	1.1 [0.1–3.1]	0.402	359 (82.1)
CC (*n* = 101)	23.3 [18.0 –34.7]	1.8 [0.4–4.0]	**<0.001**	89 (88.1)	1.8 [0.3–4.0]	0.167	84 (83.2)
LVOT (*n* = 70)	22.1 [12.8–34.9]	1.5 [0.3–2.8]	**<0.001**	57 (81.4)	1.4 [0.2–2.6]	0.330	56 (80.0)
Non-OT LV (*n* = 83)	15.1 [5.8–26.9]	1.5 [0.3–4.0]	**<0.001**	58 (70.0)	1.6 [0.3–3.8]	0.403	57 (68.7)
RVOT (*n* = 127)	20.9 [11.6–34.9]	1.2 [0.2–2.6]	**<0.001**	116 (91.3)	1.2 [0.3–2.6]	0.108	112 (88.2)
Non-OT RV (*n* = 56)	18.2 [11.2–34.2]	2.0 [0.4–2.9]	**<0.001**	52 (92.9)	2.1 [0.4–2.9]	0.345	50 (89.3)

Abbreviations: CC: coronary cusps; F.U.: follow-up, LVOT: left ventricular outflow tract, OT: outflow tract, RVOT: right ventricular outflow tract. Statistically significant values have been indicated in bold.

**Table 4:** Primary outcome predictors.

	HR [IQR]	*p*	aHR [IQR]	*p*
Sex	0.79 [0.50–1.25]	0.319		
Age (/year)	1.01 [0.98–1.02]	0.628		
Sport	0.63 [0.29–1.37]	0.244		
HF	0.95 [0.70–1.29]	0.739		
LVEF (/%)	1.01 [0.97–1.03]	0.872		
Structural heart disease	2.15 [1.37–3.38]	**<0.001**	1.96 [1.22–3.14]	**0.005**
AAD use during follow-up	1.12 [0.67–1.87]	0.656		
PVC burden	0.96 [0.94–0.97]	**<0.001**	0.96 [0.95–0.98]	**<0.001**
PVC QRS length (/ms)	0.98 [0.97–1.01]	0.118		
PVC coupling (/ms)	0.99 [0.99–1.01]	0.283		
CC origin	0.85 [0.50–1.46]	0.557		
LVOT origin	1.06 [0.60–1.90]	0.830		
Non-OT LV origin	2.60 [1.62–4.17]	**<0.001**	1.77 [1.07–2.93]	**0.027**
Non-OT RV origin	0.61 [0.35–1.07]	0.084		
RVOT origin	0.51 [0.22–1.17]	0.112		

Abbreviations: AAD: antiarrhythmic drugs, CC: coronary cusp, HF: heart failure, HR: hazard ratio, IQR: interquartile range, LVEF: left ventricular ejection fraction, LVOT: left ventricular outflow tract, PVC: premature ventricular complex, RVOT: right ventricular outflow tract. Statistically significant values have been indicated in bold.

## Data Availability

The data that support the findings of this study are available from the corresponding author, upon reasonable request.
